# Effect of Vulcanization on the Electro-Mechanical Sensing Characteristics of Multi-Walled Carbon Nanotube/Silicone Rubber Composites

**DOI:** 10.3390/polym15061412

**Published:** 2023-03-12

**Authors:** Bangwei Wan, Yang Yang, Rongxin Guo, Zhengming Fan, Peng Deng, Shibo Zhang

**Affiliations:** 1 Yunnan Key Laboratory of Disaster Reduction in Civil Engineering, Kunming 650500, China; 2 Faculty of Civil Engineering and Mechanics, Kunming University of Science and Technology, Kunming 650500, China

**Keywords:** multi-walled carbon nanotubes, silicone rubber, resistance–strain response, structural health monitoring, smart sensing composite

## Abstract

In order to realize effective monitoring for the working performance of seismic isolation structures, a multi-walled carbon nanotube (MWCNT)/methyl vinyl silicone rubber (VMQ) composite was prepared via mechanical blending using dicumyl peroxide (DCP) and 2,5-dimethyl-2,5-di(tert-butyl peroxy)hexane (DBPMH) as vulcanizing agents. The effects of the different vulcanizing agents on the dispersion of the MWCNT, electrical conductivity, mechanical properties, and resistance–strain response of the composites were investigated. The experimental results showed that the percolation threshold of the composites prepared with the two vulcanizing agents was low, while the DCP-vulcanized composites showed high mechanical properties and a better resistance–strain response sensitivity and stability, especially after 15,000 loading cycles. According to the analysis using scanning electron microscopy and Fourier infrared spectroscopy, it was found that the DCP contributed higher vulcanization activity, a denser cross-linking network, better and uniform dispersion, and a more stable damage–reconstruction mechanism for the MWCNT network during the deformation load. Thus, the DCP-vulcanized composites showed better mechanical performance and electrical response abilities. When employing an analytical model based on the tunnel effect theory, the mechanism of the resistance–strain response was explained, and the potential of this composite for real-time strain monitoring for large deformation structures was confirmed.

## 1. Introduction

Conductive polymer composite (CPC) flexible strain sensors are extensively utilized in health monitoring [1], electronic devices [2], electronic skin [3], and motion monitoring [4]. CPCs have high conductivity, strong mechanical qualities, high strain sensitivity, and outstanding deformation recovery ability [5,6,7]; notably, conductive rubber composites with extensibility [8] have quickly evolved in flexible strain sensors in recent years.

Liquid metal [9], metal nanowires [10], and nanoparticles [11] may form conductive networks within polymers. However, the cross-linking network between these conductive fillers and the polymer matrix is weak, the preparation method is complicated, the reversibility is poor, and it is difficult to match the application requirements. Since the emergence of carbon nanotubes (CNTs), their light weight, high conductivity, high strength, and high aspect ratio have made them suitable fillers for the damage sensing of polymer composites with good comprehensive performance [12,13,14,15].

CNT fillers produce a conductive network in a rubber matrix, providing the composites with conductivity and strain sensing. Xu et al. [16] used CNTs, MXene, and porous polydimethylsiloxane (PDMS) to create a multifunctional strain sensor with a 105% strain range, where the sensitivity (GF) = 1939 and the reaction speed was 158/160 ms. Li et al. [17] developed multi-walled carbon nanotube (MWCNT)/polystyrene-b-ethylene-butylene-b-styrene (SEBS) composites via wet spinning. They evaluated the sensing characteristics of the MWCNT content and aspect ratio (L/D) of the composites. The composites had a high conductivity, a strain range of 0–506%, and a GF of 197.344 when the L/D was 20/15. Despite their great sensitivity and wide detection range, these sensors are used for human physiological monitoring. Liu et al. [18] developed MWCNT/natural rubber composites using a solution technique with a percolation threshold of 3.5 wt%, a GF of 27 when the strain range was 200%, and an isolation structure strain sensor. However, it required several chemical reagents, the preparation method was complicated, and the sensitivity was poor. In conclusion, strain monitoring seismic isolation structures requires CPCs with good deformation recovery, high sensitivity, and simple preparation process.

Silicone rubber is utilized in sealing [19], electronic equipment [20], and medicine [21]. Methyl vinyl silicone rubber (VMQ) has a highly saturated structure and low curing activity, making it unsuitable for traditional curing systems. Organic peroxide is a typical vulcanizing agent. The selection of VMQ vulcanizing agent is crucial [22,23,24]. Commonly used vulcanizing agents are Dicumyl peroxide (DCP) and 2,5-Dimethyl-2,5-di(tert-butyl peroxy)hexane (DBPMH). Chatteriee et al. [25] improved char safety by mixing the inhibitor 2,2,6,6-Tetramethylpiperidine 1-oxyl (TEMPO) with DCP. This prevented the VMQ from creating peroxide free radicals during vulcanization. Liu et al. [26] employed carbon black as a filler and DCP as a vulcanizing agent to evaluate the effects of dosage and curing time on VMQ composites’ electrical conductivity and mechanical qualities. Ma et al. [27] studied the mechanical characteristics, radiation resistance, and thermal aging resistance of DBPMH on VMQ and methyl phenyl silicone rubber (MPSR) blends at various temperatures. Lin et al. [28] found that DBPMH was the best UV-A-resistant vulcanizing agent for VMQ degradation. However, the preceding research mostly concentrated on VMQ mechanical characteristics, aging resistance, and searing resistance. VMQ strain-sensing characteristics are seldom reported. Thus, studying how various vulcanizing agents affect VMQ strain-sensing characteristics is essential.

This study used MWCNT as a conductive filler, SiO_2_ as a reinforcing filler, and VMQ as a matrix. The MWCNT/VMQ composites were made via mechanical blending using DCP and DBPMH as vulcanizing agents. The conductive network shape and interface interaction of the two types of vulcanizing agents on the conductive nanocomposites were researched, and the effects of different strains and rates on the resistance–strain response performance under cyclic load was investigated. A mechanical and electrical performance analysis model for the entire cycle of loading and unloading was developed. This work will aid CPC structural strain monitoring during large deformations.

## 2. Experimental

### 2.1. Materials

Methyl vinyl silicone rubber (VMQ, grade: 110-2, density of 1.1 g/cm^3^, and a molecular weight of 6.2 × 10^5^ g/mol, Nanjing Dongjue Silicone Co., Ltd., Nanjing, China), Multi-walled carbon nanotubes (MWCNT with lengths of 10–20 μm, outer diameters of 4~6 nm, specific surface areas of 500~700 m^2^ g^−1^, and purities of >98%, China Organic Chemical Co., Ltd., Chinese Academy of Sciences, Chengdu, China), Dicumyl peroxide (DCP, white granule, density of 1.026 g/cm^3^_,_ Qiangsheng Chemical Co., Ltd., Suzhou, China), 2,5-Dimethyl-2,5-di(tert-butyl peroxy)hexane (DBPMH, pale yellow oily liquid, density of 0.8650 g/cm^3^_,_ Shanghai McLean Biochemical Technology Co., Ltd., Shanghai, China), Silicon dioxide (SiO_2_, with purity of > 99.8%, BET of 300 m^2^/g, and a particle size of 7–40 nm, Shanghai McLean Biochemical Technology Co., Ltd., Shanghai, China), Hydroxy silicone oil (HPMS, colorless transparent liquid, density of 0.98 g/cm^3^, Anhui Aita Silicone Oil Co., Ltd., Bengbu, China).

### 2.2. Preparation of the MWCNT/VMQ Composites

**Figure 1:**
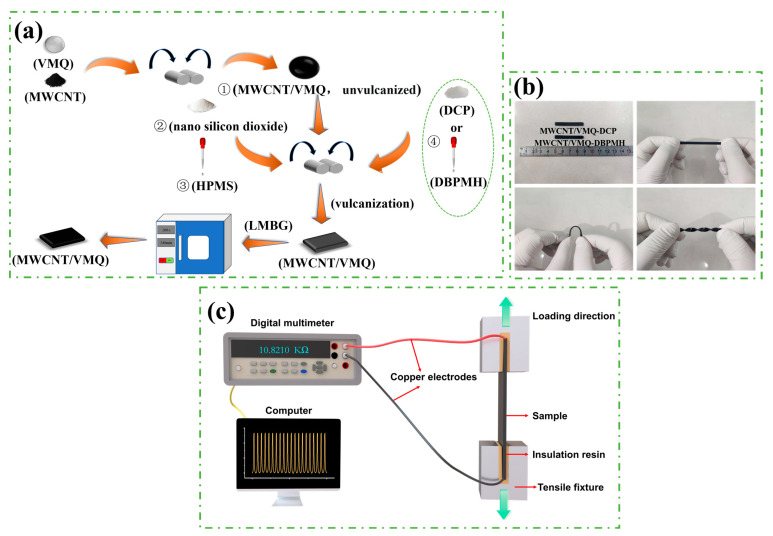
(**a**) Preparation process of the conductive silicone rubber composites, ①–④ indicates the adding sequence. (**b**) Image of the specimen under original, tensile, bending and torsion conditions. (**c**) Device and test used to determine the strain sensing behavior of the composites.

The MWCNT/VMQ composites were prepared via mechanical blending. The preparation process is shown in Figure 1a and the experimental formula is shown in Table 1. First, we put the VMQ into a twin-roll mixer (roll pitch of 1 mm and rotating speed of 17 r/min at room temperature), conducted a narrow pass, and slowly added MWCNT into the VMQ to make it mix evenly. Then, we reduced the roll pitch, successively adding SiO_2_ and HPMS, and further opened the mill to mix the materials evenly. DCP was then added to the composites and then the mixture was placed in a rectangular vulcanization mold at 170 °C for 10 min to obtain the conductive silicone rubber composites. The vulcanized composites were dried for 4 h at 200 °C under normal pressure. The prepared composites were recorded as MWCNT/VMQ-DCP. The DBPMH vulcanized composites MWCNT/VMQ-DBPMH was prepared by the same method. Figure 1b shows the photos of the composites under original, tensile, bending, and torsion conditions.

### 2.3. Characterization

The dispersion state of the MWCNT in the VMQ matrix was observed by FE-SEM (Nova Nano SEM450, Hillsboro, OR, USA) using a scanning electron microscope. The composites were cleaned with anhydrous ethanol, and then the composites were made brittle in liquid nitrogen and the platinum was sprayed in a vacuum. The interface interaction between the MWCNT and the VMQ was analyzed by FT-IR using attenuated total reflection Fourier transform infrared spectroscopy (Bruker Tensor 27, Billericay Town City, Germany) with a wavenumber range of 600–3200. We used an Agilent 34410 digital multimeter (Keysight Technology, Santa Rosa, CA, USA) to measure the resistance of the composites. The composites were cut into 40 mm × 40 mm × 1 mm strips, taking a 90 s resistance value as the average value. According to the conductivity Formula (1) used for calculating the volume conductivity, σ (S/cm) is expressed as:

(1) $$\begin{array}{c} \sigma =\frac{1}{\rho }=\frac{L}{RS}, \end{array}$$

where *ρ* is the volume resistivity (Ω·cm) of the sample, *R* is the volume resistance (Ω), *L* is the length (cm) of the sample, and *S* is the cross-sectional area (cm^2^). The mechanical property test was carried out on a DDL10 electronic universal testing machine (China Changchun Testing Machine Research Institute Co., Ltd., Changchun, China). The tensile rate was 200 mm/min, and the average of the three groups of experimental data was taken. We fixed the sample on the electronic universal testing machine to carry out the cyclic loading–unloading test, and we used a digital multimeter to record the changes in the electrical signals in real time. The schematic diagram of the experimental test device is shown in Figure 1c.

## 3. Results and Discussion

### 3.1. MWCNT Dispersion Analysis and Interface Effect of the Two Composites

**Figure 2:**
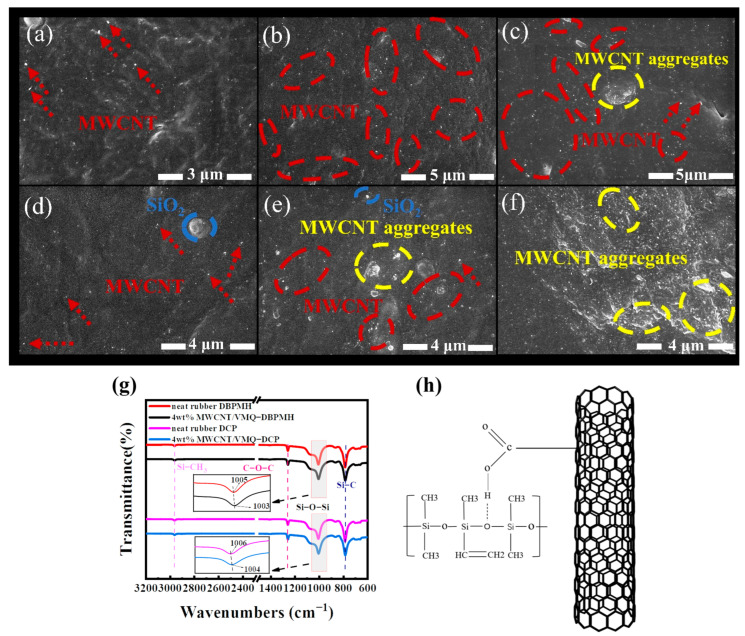
(**a**–**f**) SEM images of the MWCNT/VMQ-DCP and MWCNT/VMQ-DBPMH composites with different MWCNT mass fractions. MWCNT/VMQ-DCP: (**a**) 2 wt% MWCNT, (**b**) 3 wt% MWCNT. (**c**) Images of 6 wt% MWCNT. MWCNT/VMQ-DBPMH, (**d**) 2 wt% MWCNT. (**e**) Image of 3 wt% MWCNT. (**f**) Image of 6 wt% MWCNT. The dark phase represents the VMQ matrix, red arrow, and bright spot, and the red circle represents the MWCNT cross-section, the blue circle represents the SiO_2_, and the yellow circle represents the MWCNT aggregates. (**g**) FT-IR spectra of the neat VMQ and 4 wt% MWCNT composites with different vulcanizing agents. (**h**) The hydrogen bond that formed between the VMQ matrix and the MWCNT.

The MWCNT dispersion in the VMQ matrix affected the composites’ conductivity and resistance–strain response. Figure 2 shows the microscopic morphologies of the MWCNT/VMQ-DCP and MWCNT/VMQ-DBPMH composites. As shown in Figure 2a,d, the MWCNT in the two composites was sparse and did not form a conductive network at 2 wt%. The MWCNT in the MWCNT/VMQ-DBPMH composites grew dramatically at 3 wt%, forming visible aggregates, as shown in Figure 2e. As demonstrated in Figure 2b, the MWCNT/VMQ-DCP composites were evenly dispersed in the VMQ matrix and formed a conductive network. Figure 2c,f show that the MWCNT/VMQ-DCP composites contained fewer aggregates and were more equally distributed than the MWCNT/VMQ-DBPMH composites at 6 wt%. The MWCNT and VMQ in the DCP-vulcanization products were compatible and well-dispersed.

**Figure 3:**
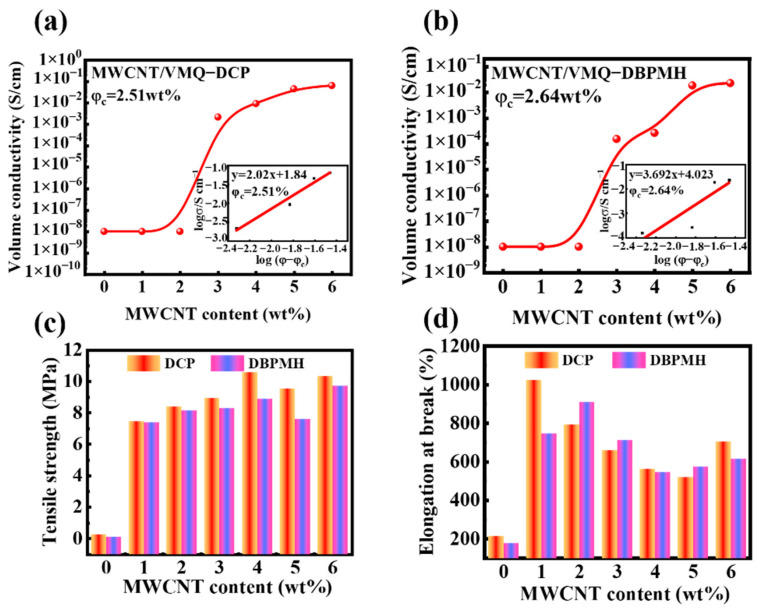
(**a**) Volumetric conductivity of the MWCNT/VMQ-DCP composites. (**b**) Volumetric conductivity of the MWCNT/VMQ-DBPMH composites. (**c**) Tensile strengths of the two composites with different vulcanizing agents. (**d**) Elongation breaks of the two composites with different vulcanizing agents.

FT-IR was used to explain the composites’ interface interactions. Figure 2g shows the spectra of the neat VMQ and MWCNT/VMQ composites under various vulcanizing agents. The Si-CH_3_ and Si-O stretching vibrations produced peaks at 2962 cm^−1^ and 699 cm^−1^. The C-O-C bending vibration caused the 1257 cm^−1^ peak. The Si-O-Si telescopic vibration caused peaks in the range of 1003–1006 cm^−1^. In addition, from the partial enlargement of Figure 2g, we can see that the characteristic peak of MWCNT/VMQ-DBPMH composites moved from 1005 cm^−1^ to 1003 cm^−1^, while the characteristic peak of the MWCNT/VMQ-DCP composites moved from 1006 cm^−1^ to 1004 cm^−1^. This was due to the hydrogen bonds between several of the carboxyl groups on the MWCNT surface and the Si-O-Si groups in the VMQ, as shown in Figure 3b, which promoted compatibility.

### 3.2. Mechanical Properties and Conductivity Properties of the Two Composites

Figure 3a,b show the MWCNT/VMQ-DCP and MWCNT/VMQ-DBPMH composites’ volume conductivities. The two composites measured volume conductivities increased gradually with the MWCNT concentration. Both composites showed obvious seepage phenomena [29]. The percolation theory [30] uses the following equation to investigate the link between composite conductivity and MWCNT content:

(2) $$\sigma =\sigma_{0}{\left(\varphi -\varphi_{c}\right)}^{t},$$

where *σ* is the volume conductivity of the composites at a certain nanofiller content, *σ*_0_ is the scale factor, *φ* is the mass fraction of the nanofillers in the conductive composites, *φ_c_* is the percolation threshold of the conductive composites, and *t* is the critical resistivity factor, which is related to the dimensions of the conductive networks. The two-dimensional and three-dimensional conductive network dimensions are *t* = 1.2 and *t* = 2, respectively [31,32]. The MWCNT/VMQ-DCP and MWCNT/VMQ-DBPMH composites had 2.51 wt% and 2.64 wt% percolation thresholds, respectively. The linear relationships between the fitted data produced *t* = 2.02 and *t* = 3.692. The two composites may have formed a tunneling conductive network because their t values were greater than that of the three-dimensional conductive network dimension [33].

Strain-sensing materials in engineering require good mechanical characteristics. Figure 3c demonstrates that the neat VMQ rubber had poor mechanical characteristics and a tensile strength of 0.35 MPa. Adding SiO_2_ and MWCNT increased the tensile strength to 10.57 MPa. The cross-linking interaction between the organic chain of the SiO_2_ and the polymer chain of the VMQ rubber inhibited the mobility of the VMQ molecular chain, boosting the effective cross-linking density and the tensile strength [34]. The synergistic effects of SiO_2_ and MWCNT established an effective stress transmission between the VMQ matrix and the two fillers [35]. In addition, when the content of MWCNT was in the range of 3–6%, the tensile strength of the DCP vulcanizate was significantly higher than that of the DBPMH vulcanizate. This is because DCP has higher activity and a higher degree of vulcanization, resulting in a higher degree of cross-linking in the VMQ three-dimensional molecular network [36]. Figure 3d shows the change in the elongation at the break of the two composites. Both composites had low break elongations without fillers. The SiO_2_ and MWCNT quickly enhanced the two composites’ break elongations. With the increase in MWCNT content, the elongation at the break began to decrease. The aggregation of MWCNT and SiO_2_ in the VMQ matrix destroyed the matrix continuity and produced flaws, reducing the elongation at the break. When the MWCNT concentration was in the range of 5–6%, the elongation at the break increased again because the MWCNT dispersed the stress in the matrix, compensating for the aggregation-induced mechanical characteristic loss [37].

### 3.3. Resistance–Strain Response Characteristics of the Composites

#### 3.3.1. Strain Response under Static Loads

**Figure 4:**
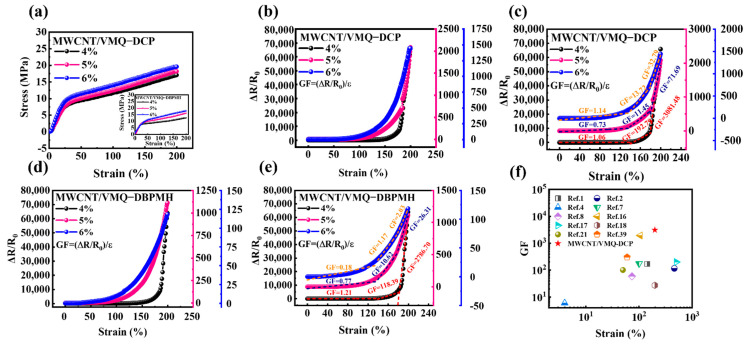
(**a**) Representative stress–strain curves of the two composites with various MWCNT contents. (**b**) The relationship between the strain and the resistance of the MWCNT/VMQ-DCP composite with different MWCNT contents. (**c**) The relationship between the sensitivity coefficient and the strain of the MWCNT/VMQ-DCP composites with different MWCNT contents. (**d**) The relationship between the strain and the resistance of the MWCNT/VMQ-DBPMH composite with different MWCNT contents. (**e**) The relationship between the sensitivity coefficient and the strain of the MWCNT/VMQ-DBPMH composites with different MWCNT contents. (**f**) The GF values and strain-sensing ranges of the MWCNT/VMQ-DCP composites.

Figure 4a shows the strain-stress curves at 4 wt%, 5 wt%, and 6 wt% MWCNT content, with a maximum strain of 200%. Obviously, when strain increases, stress increases and the composites deform, the two composites’ resistances increased with the strain, the composites’ conductive nanofiller spacing rose as the strain increased, reducing electron hopping, destroying the conductive network, and increasing the resistance [1], as shown in Figure 4b,d. Compared to Figure 4b,d, the MWCNT/VMQ-DCP composites’ resistance maximum (65,869) was higher than that of the MWCNT/VMQ-DBPMH composites (63,952), showing a stronger strain sensitivity. The resistance response mechanisms varied by strain stage. The linear and exponential response areas were 0–100% and 100–200%, respectively. The MWCNT network only deformed locally in the linear response region, and it expanded slowly. The exponential response zone destroyed the MWCNT conductive network, increasing the resistance [38].

For the MWCNT/VMQ-DCP and MWCNT/VMQ-DBPMH composites resistance–strain sensitivity evaluation, GF = $(\Delta R/R_{0})/\varepsilon$, where ΔR is the resistance change value of the test piece and $R_{0}$ is the initial resistance ε for the strain. Figure 4c,e show the change in GF values for the two composites. The GF value rose with the strain, suggesting that the higher the strain, the more sensitive the reaction. When the MWCNT content was 4 wt%, the composites were very sensitive. MWCNT/VMQ-DCP has a maximum GF value of 3081.48 and MWCNT/VMQ-DBPMH has a maximum GF value of 2786.7. The DCP-vulcanized materials were more sensitive. The maximum GF values and strain ranges of the strain sensors in recent years are shown in Figure 4f. In contrast, this study achieved a higher resistance–strain response sensitivity.

#### 3.3.2. Resistance–Strain Response under Cyclic Load

**Figure 5:**
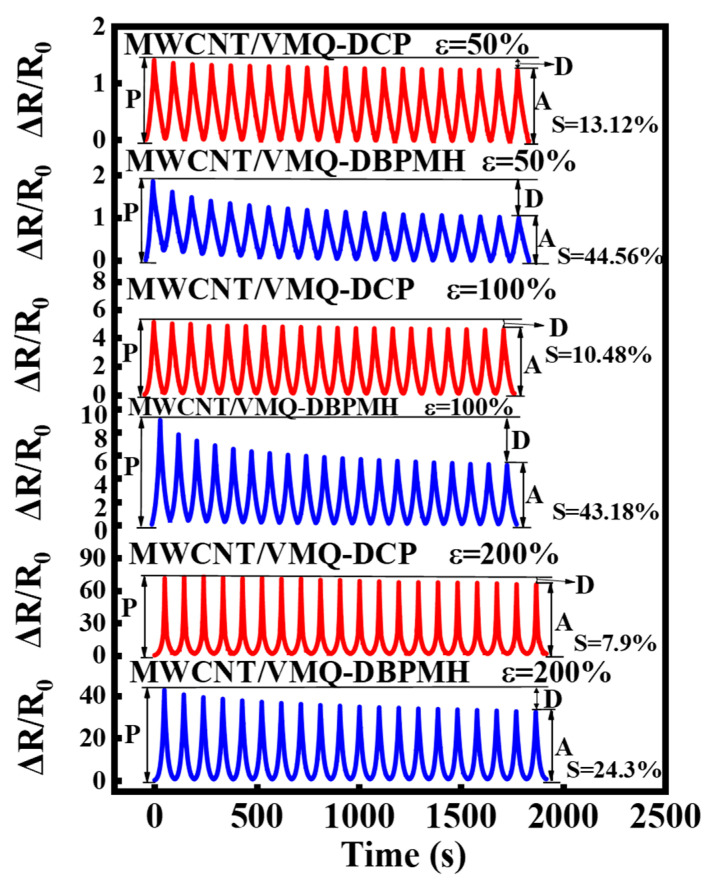
The resistance of the MWCNT/VMQ-DCP composites and the MWCNT/VMQ-DBPMH composites under different levels of cyclic strain and the resistivity changes in the two composites under different strain cycles.

Figure 5 illustrates the two composites’ cyclic resistance–strain responses at 50%, 100%, and 200% strains when the content of MWCNT was 4 wt%. The MWCNT/VMQ-DBPMH composites resistance decreased and stabilized with the cycles. This was most noticeable at 50% strain. MWCNT/VMQ-DCP composites have always had steady resistance. The DCP-vulcanized material MWCNT had better dispersion, a more stable conductive network structure, and a more stable resistance–strain response, and showed the ratio between the decrease in peak value during cyclic strains (D) and the resistivity peak of the first cycle (P) and the amplitude of the peak resistivity (A). The formula S=DP indicates the recovery rate of the conductive network under cyclic loading and unloading. As the ratio increased, the conductive network recovered less well. The DCP-vulcanized material had better conductive network recovery.

**Figure 6:**
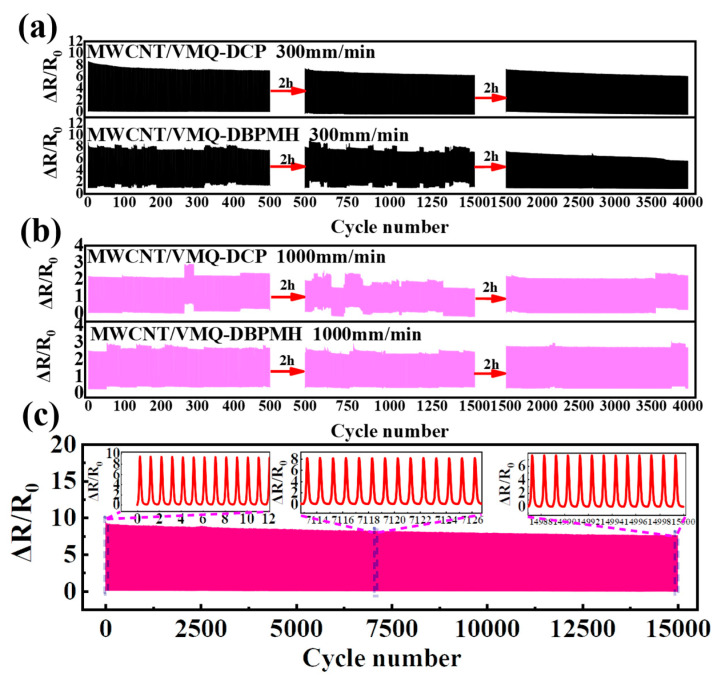
(**a**) The resistance of the MWCNT/VMQ-DCP composites and the MWCNT/VMQ-DBPMH composites under 300 mm/min rates of cyclic strain. (**b**) The resistance of the MWCNT/VMQ-DCP composites and the MWCNT/VMQ-DBPMH composites under 1000 mm/min rates of cyclic strain. (**c**) The resistance of the MWCNT/VMQ-DCP composites under a strain of 15,000 cycles.

Figure 6a,b illustrate the resistance–strain response of the two composites under 4000 cyclic loads at 300 and 1000 mm/min when the content of MWCNT was 4 wt%. The experiment included three phases with 500, 1000, and 2500 cyclic loading cycles with a 2 h intermittent period. The MWCNT/VMQ-DBPMH composite’s electrical signals fluctuated throughout the first 1500 cycles at 300 mm/min. After repeated destruction–reconstruction, the network structure became stable, and the resistance response was also stable. This did not occur with the DCP-vulcanized composites. At 1000 mm/min, the electrical signals from the two composites exhibited resistance response signal instability. The resistance signal was unstable because, as the strain frequency increased, the conductive network of the two composites broke, and reconstruction was difficult to keep up with, resulting in significant damage and difficulty with reconstruction after many cycles. Accordingly, the MWCNT/VMQ-DCP composites were loaded 15,000 times. As demonstrated in Figure 6c, the MWCNT/VMQ-DCP composite’s resistance–strain response remained steady without shoulder peaks. Compared to other rubber composites, it has obvious advantages [1,18,39], indicating that the DCP-vulcanized agent helped preserve the sensor signals stability.

#### 3.3.3. Strain-Sensing Mechanism

**Figure 7:**
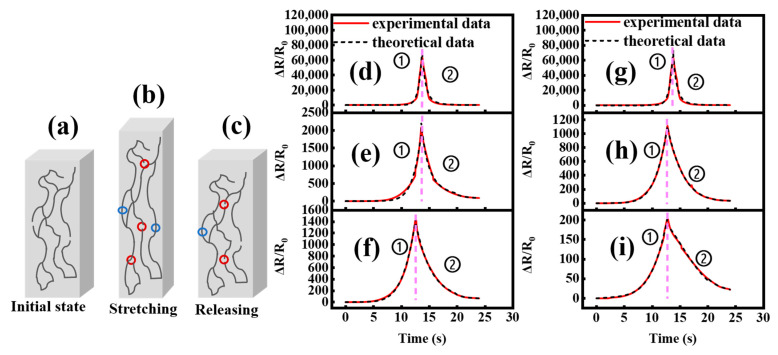
(**a**–**c**) MWCNT conductive network changes in the whole cycle, where disconnection is represented by the red ellipse and recombination is shown with the blue ellipse. The MWCNT/VMQ-DCP at MWCNT content of (**d**) 4 wt%, (**e**) 5 wt%, and (**f**) 6 wt%, and the MWCNT/VMQ-DBPMH at MWCNT content of (**g**) 4 wt%, (**h**) 5 wt%, and (**i**) 6 wt%, with ① denoting the tensile stage and ② denoting unloading (theoretical predictions are shown in black and experimental results are shown in red).

The interpretation and quantitative analysis of composites resistance–strain response mechanisms are required for strain monitoring sensors. Figure 7 illustrates the composites’ MWCNT conductive network during tension unloading. The initial state of the MWCNT in the VMQ matrix is shown in Figure 7a. Figure 7b shows that when strain grew, some cross-linking points in the network were disconnected, the conductive path was reduced, and resistance increased. The severed cross-linking point reconnected, the conductivity rose, and the resistance fell to the starting test result when the load was removed, as illustrated in Figure 7c. Thus, the deformation and destruction–reconstruction of the conductive network caused a synchronized resistance–strain response cycle. This model quantifies resistance–strain relationships. According to the applicable theory [1], resistance rises with strain at the tensile stage as shown below:

(3) $$\begin{array}{c} N_{1}=N_{0}/1+{\left(\frac{\varepsilon }{\varepsilon_{c}}\right)}^{2m}, \end{array}$$

(4) $$\begin{array}{c} \rho \propto {\left(N\right)}^{{-n}_{\varepsilon }},\mathrm{and} \end{array}$$

(5) $$\begin{array}{c} \frac{\Delta R}{R_{0}}=\frac{\rho }{\rho_{0}}{\left(\varepsilon +1\right)}^{2}-1={\left(\varepsilon +1\right)}^{2}{\left[{\left(1+{\left(\frac{\varepsilon }{\varepsilon_{c}}\right)}^{2m}\right)}^{-1}\right]}^{{-n}_{\varepsilon }}-1, \end{array}$$

where $N_{1}$ is the number of inter-particle connections per volume in the process of stretching, $N_{0}$ is the initial number of inter-particle connections per volume, m is a constant related to the fractal structure of the network, $\varepsilon_{c}$ is a constant which can be interpreted as the yield strain, and $n_{\varepsilon }$ is a scaling exponent. In the releasing process, the breakage and destruction of the conductive network exist simultaneously. Some damaged conductive networks are irreversible, leading to an increase in resistance compared to the initial value. The following equation is used to describe the change in the inter-particle connections number $N_{2}$ due to the complex reformation of the inter-particle connections and the irreversible conductive networks in the releasing stage:

(6) $$\begin{array}{c} N_{2}\left(t\right)=N_{0}\left(k_{1}-k_{2}e^{-\mathrm{Kt}}\right)=N_{0}\left(k_{1}-k_{2}e^{-K\frac{\varepsilon }{\dot{\varepsilon }}}\right), \end{array}$$

where $k_{1},k_{2},\;\mathrm{and}\;K$ are constants associated the re-formation process of the inter-particle connections and $\dot{\varepsilon }$ is the strain rate. Based on Equations (4)–(6), $\frac{\Delta R}{R_{0}}$ in the releasing stage is:

(7) $$\begin{array}{c} \frac{\Delta R}{R_{0}}={\left(\varepsilon +1\right)}^{2}{\left[{\left(1+{\left(\frac{\varepsilon }{\varepsilon_{c}}\right)}^{2m}\right)}^{-1}+k_{1}-k_{2}e^{-K\frac{\varepsilon }{\dot{\varepsilon }}}\right]}^{-n_{\varepsilon }}-1. \end{array}$$

The model shows the cycle’s resistance–strain connection. The experimental results and theoretical predictions of the two composites’ resistances throughout the cycle are displayed in Figure 7d–i, and the relevant fitting parameters are reported in Table 2 and Table 3

For the MWCNT/VMQ composites with different vulcanizing agents, the $n_{s}/\varepsilon_{c}$ decreased continuously with the increase in MWCNT content, which was consistent with the change in GF value. The change in m implied that the two composites’ conductive networks differed significantly. The reconnection process between the conductive network structure particles was affected by $k_{1},\;k_{2},$ and K, resulting in variable resistance–strain response performances.

## 4. Conclusions

In order to produce strain-monitoring sensing materials for large deformation structures, the preparation techniques, microstructures, electro-mechanical properties, and dynamic resistance–strain response characteristics of multi-walled carbon nanotubes (MWCNT)/methyl vinyl silicone rubber (VMQ) composites vulcanized with DCP and DBPMH were systematically studied. Our results were as follows:

(1) The MWCNT/VMQ-DCP and VMQ-DBPMH composites had 2.51 wt% and 2.64 wt% percolation thresholds, respectively. Both composites had MWCNT three-dimensional tunneling conductive networks;
(2) Under the synergistic effects of MWCNT and SiO_2_, the DCP high activity increased the VMQ cross-linking and the composites’ mechanical characteristics;
(3) Compared to the DBPMH-vulcanized composites, the DCP-vulcanized composites had high resistance–strain response sensitivities and excellent response signal stabilities. This makes them better materials for strain-monitoring and sensing materials;
(4) The analytical model of the composites’ resistance–strain responses described the quantitative connection between resistance and strain during the tensile and unloading phases, which will guide large-strain-monitoring and the analysis of seismic isolation structures.

## Figures and Tables

**Table 1:** The formula of the MWCNT/VMQ composites (wt%).

Materials	Content
VMQ	100
MWCNT	Variable X (X = 0,1,2,3,4,5,6)
SiO_2_	40
HPMS	4
DCP	1
DBPMH	1

**Table 2:** $\Delta R/R_{0}$ parameters after strain curve fitting for the different MWCNT/VMQ-DCP composites.

MWCNT Content	m	$\varepsilon_{c}$	$n_{s}$	$k_{1}$	$k_{2}$	k
4 wt%	11.11	8.32	−3.85	1.49	2.84	1.77
5 wt%	2.94	6.89	−2.61	1.11	29.76	3.72
6 wt%	2.35	2.19	−1.94	0.49	40,451.6	11.31

**Table 3:** $\Delta R/R_{0}$ parameters after strain curve fitting for the different MWCNT/VMQ-DBPMH composites.

MWCNT Content	m	$\varepsilon_{c}$	$n_{s}$	$k_{1}$	$k_{2}$	k
4 wt%	7.79	8.57	−2.74	1.01	1.86	0.62
5 wt%	4.44	5.82	−2.65	1.47	0.21	−1.11
6 wt%	2.81	3.44	−2.74	1.17	8.44	2.61

## Data Availability

The data presented in this study are available from the corresponding author upon reasonable request.

## References

[1] Yang H., Yao X., Zheng Z., Gong L., Yuan L., Yuan Y., Liu Y. (2018). Highly sensitive and stretchable graphene-silicone rubber composites for strain sensing. Compos. Sci. Technol..

[2] Xu Y., Xie X., Huang H., Wang Y., Yu J., Hu Z. (2020). Encapsulated core–sheath carbon nanotube–graphene/polyurethane composite fiber for highly stable, stretchable, and sensitive strain sensor. J. Mater. Sci..

[3] Guo Y., Wei X., Gao S., Yue W., Li Y., Shen G. (2021). Recent Advances in Carbon Material-Based Multifunctional Sensors and Their Applications in Electronic Skin Systems. Adv. Funct. Mater..

[4] Liu X., Ren Z., Liu F., Zhao L., Ling Q., Gu H. (2021). Multifunctional Self-Healing Dual Network Hydrogels Constructed via Host–Guest Interaction and Dynamic Covalent Bond as Wearable Strain Sensors for Monitoring Human and Organ Motions. ACS Appl. Mater. Interfaces.

[5] Oh S., Kim J., Chang S.T. (2018). Highly sensitive metal-grid strain sensors via water-based solution processing. RSC Adv..

[6] Liu L., Gao Y., Liu Y., Xu M., Yang S., Li K., Zhao S., Cao D., Ahn J.H. (2022). Biomimetic metal–organic framework-derived porous carbon welded carbon nanotube networks for strain sensors with high sensitivity and wide sensing range. Appl. Surf. Sci..

[7] Christ J.F., Aliheidari N., Ameli A., Pötschke P. (2017). 3D printed highly elastic strain sensors of multiwalled carbon nanotube/thermoplastic polyurethane nanocomposites. Mater. Design..

[8] Liu Y., Kumar S. (2014). Polymer/carbon nanotube nano composite fibers—A review. ACS Appl. Mater. Interfaces.

[9] Yan J., Malakooti M.H., Lu Z., Wang Z., Kazem N., Pan C., Bockstaller M.R., Majidi C., Matyjaszewski K. (2019). Solution processable liquid metal nanodroplets by surface-initiated atom transfer radical polymerization. Nat. Nanotechnol..

[10] Min S.H., Lee G.Y., Ahn S.H. (2019). Direct printing of highly sensitive, stretchable, and durable strain sensor based on silver nanoparticles/multi-walled carbon nanotubes composites. Compos. Part B Eng..

[11] Kim Y., Zhu J., Yeom B., Di Prima M., Su X., Kim J.G., Yoo S.J., Uher C., Kotov N.A. (2013). Stretchable nanoparticle conductors with self-organized conductive pathways. Nature.

[12] Sam-Daliri O., Faller L.-M., Farahani M., Roshanghias A., Araee A., Baniassadi M., Oberlercher H., Zangl H. (2019). Impedance analysis for condition monitoring of single lap CNT-epoxy adhesive joint. Int. J. Adhes. Adhes..

[13] Ahmed S., Schumacher T., Thostenson E.T., McConnell J. (2020). Performance Evaluation of a Carbon Nanotube Sensor for Fatigue Crack Monitoring of Metal Structures. Sensors.

[14] Lim A.S., Melrose Z.R., Thostenson E.T., Chou T.-W. (2011). Damage sensing of adhesively-bonded hybrid composite/steel joints using carbon nanotubes. Compos. Sci. Technol..

[15] Sam-Daliri O., Faller L.-M., Farahani M.H., Zangl (2021). Structural health monitoring of adhesive joints under pure mode I loading using the electrical impedance measurement. Eng. Fract. Mech..

[16] Xu B., Ye F., Chen R., Luo X., Chang G., Li R. (2022). A wide sensing range and high sensitivity flexible strain sensor based on carbon nanotubes and MXene. Ceram. Int..

[17] Li L., Du Z., Sun B., Li W., Jiang L., Zhou Y., Ma J., Chen S., Zhou F.L. (2022). Fabrication of electrically conductive poly(styrene-b-ethylene-ran-butylene-b-styrene)/multi-walled carbon nanotubes composite fiber and its application in ultra-stretchable strain sensor. Eur. Polym. J..

[18] Liu X., Guo R., Lin Z., Yang Y., Xia H., Yao Z. (2021). Resistance-strain sensitive rubber composites filled by multiwalled carbon nanotubes for structuraldeformation monitoring. Nanomater. Nanotechnol..

[19] Yang H., Yao X.F., Yan H., Yuan Y.N., Dong Y.F., Liu Y.H. (2017). Anisotropic hyper-viscoelastic behaviors of fabric reinforced rubber composites. Compos. Struct..

[20] Du S., Zhang Y., Meng M., Tang A., Li Y. (2021). The role of water transport in the failure of silicone rubber coating for implantable electronic devices. Prog. Org. Coat..

[21] Azizkhani M.B., Rastgordani S., Anaraki A.P., Kadkhodapour J., Hadavand B.S. (2020). Highly sensitive and stretchable strain sensors based on chopped carbon fibers sandwiched between silicone rubber layers for human motion detections. J. Compos. Mater..

[22] Zhang Y.X., Li B. (2014). Effect of Electrical Conductivity and Physical Performance about Vulcanization System in Conductive Silicon Rubber. Appl. Mech. Mater..

[23] Chatterjee T., Wiessner S., Naskar K., Heinrich G. (2016). Exploring a novel cyclic monofunctional peroxide for curing of silicone rubber at elevated temperature. Polym. Eng. Sci..

[24] Hudec I., Sýkora R., Kruželák J. (2017). Vulcanization of Rubber Compounds with Peroxide Curing Systems. Rubber Chem. Technol..

[25] Chatterjee T., Wiessner S., Naskar K., Heinrich G. (2014). Novel thermoplastic vulcanizates (TPVs) based on silicone rubber and polyamide exploring peroxide cross-linking. Express Polym. Lett..

[26] Liu X., Chen C., Yang Y. (2012). Preparation and Performance of Silicone Rubber Composite Filled with Carbon Black. Material.

[27] Ma D., Tan J., Li Y., Bian C., Wang G., Feng S. (2014). Curing characteristics, morphology, thermal stability, mechanical properties, and irradiation resistance of methylethylsilicone/methylphenylsilicone rubber blends. J. Appl. Polym. Sci..

[28] Lin Y., Yin F., Liu Y., Wang L., Wu K. (2020). Influence of vulcanization factors on UV-A resistance of silicone rubber for outdoor insulators. IEEE Trans. Dielectr. Electr. Insul..

[29] Lu Y., Wu H., Liu J., Liu Y., Zhao J., Liu P., Wang J., Liang H., Huang Y., Song A. (2019). Electrical percolation of silicone rubber filled by carbon black and carbon nanotubes researched by the Monte Carlo simulation. J. Appl. Polym. Sci..

[30] Huang J.-C. (2002). Carbon black filled conducting polymers and polymer blends. Adv. Polym. Technol..

[31] Surve M., Pryamitsyn V., Ganesan V. (2006). Universality in Structure and Elasticity of Polymer-Nanoparticle Gels. Phys. Rev. Lett..

[32] Bauhofer W., Kovacs J.Z. (2009). A review and analysis of electrical percolation in carbon nanotube polymer composites. Compos. Sci. Technol..

[33] Deng H., Bilotti E., Zhang R., Loos J., Peijs T. (2010). Effect of thermal annealing on the electrical conductivity of high-strength bicomponent polymer tapes containing carbon nanofillers. Synth. Met..

[34] Wu C., Gao Y., Liang X., Gubanski S.M., Wang Q., Bao W., Li S. (2019). Manifestation of Interactions of Nano-Silica in Silicone Rubber Investigated by Low-Frequency Dielectric Spectroscopy and Mechanical Tests. Polymers.

[35] Tan C., Liu G., Yao H., Li X., Li G., Qing L., Yang Y. (2020). Mechanical, dielectric, and thermal properties of fluorosilicone rubber composites filled with silica/multiwall carbon nanotube hybrid fillers. J. Appl. Polym. Sci..

[36] Baquey G., Moine L., Degueil-Castaing M., Lartigue J.C., Maillard B. (2010). Decomposition of Di-tert-butyl Peroxide in Siloxane: An Approach of the Free Radical Cross-Linking of Silicones. Macromolecules.

[37] Kong J., Tong Y., Sun J., Wei Y., Thitsartarn W., Jayven C.C.Y., Muiruri J.K., Wong S.Y., He C. (2018). Electrically conductive PDMS-grafted CNTs-reinforced silicone elastomer. Compos. Sci. Technol..

[38] Rui Z., Baxendale M., Peijs T. (2007). Universal resistivity–strain dependence of carbon nanotube/polymer composites. Phys. Rev. B.

[39] Fan Z., Guo R., Yang Z., Yang Y., Liu X. (2022). The Effect of the Co-Blending Process on the Sensing Characteristics of Conductive Chloroprene Rubber/Natural Rubber Composites. Polymers.

